# Theoretical and practical aspects in total uncemented hip arthroplasty by using short femoral stem prosthesis

**Published:** 2015

**Authors:** M Moga, ME Pogarasteanu, A Barbilian

**Affiliations:** * ”Dr. Carol Davila” Central University Emergency Military Hospital, Orthopaedics–Traumathology Clinic, Bucharest, Romania

**Keywords:** hip arthroplasty, short stem, minimally invasive

## Abstract

Hip arthrosis, primary or secondary, is an osteoarthritic degenerative process that affects the hip joint. Primary hip arthrosis has an unknown etiology, and secondary hip arthrosis has well defined causes; of these causes, some are known to lead to arthrosis of the hip in the young age patient. The surgical treatment aims either to preserve the patient’s hip joint, or to replace the joint. The most commonly used procedure at this time is the total hip arthroplasty. The femoral component may have a short or a long stem. The short femoral stem prosthesis is usually impacted by using a unique technique and unique instruments, according to the manufacturer’s specifications. There are several models of short stem femoral prosthesis, but no matter which one is chosen, the surgical indication, the surgical technique and a well-conducted recovery program are important. The choosing of each arthroplastic implant must be made with care, taking into consideration the patient’s benefit, his expectations, and also the surgeon’s experience.

## Introduction

Osteoarthritis consists of a mechanical and biological disturbance in the natural process of synthesis and destruction of chondrocytes in the joint cartilage, of extracellular matrix, and of subchondral bone. The changes that lead to an articular cartilage damage include an increase in water content, the alteration of the collagen matrix, and a decrease in the proteoglycan content.

Hip arthrosis, primary or secondary, is an osteoarthritic degenerative process that affects the hip joint. Primary arthrosis has an unknown cause, affects multiple joints, and is rarely diagnosed before the age of 35, while secondary arthrosis is usually a monoarticular pathology, with a well determined cause. The causes of secondary arthrosis are the following: congenital anomaly, growth cartilage separation, mechanical instability, local trauma, and fracture [**1**].

Concerning the pathology’s evolution, there is a great difference between primary and secondary arthrosis: in primary arthrosis, the symptoms have a slow and unpredictable progression towards joint degradation, over a long period of time, having a chance of preserving the hip function for an extensive period before operating, while secondary arthrosis evolves much faster, and non-surgical treatment is of little value [**2**].

Out of all the causes for secondary hip arthrosis, some have a greater chance to lead to its appearance in the young patient.

One of these causes is congenital hip dysplasia, with an incidence of 1-1,5/ 1000 live newborns, and 80% of these cases are females [**3**]. The issues raised by this pathology include hip anatomy distortion, limb length discrepancy, and postoperative neuropathy. There can be acetabular dysplasia or femoral dysplasia, and it has a multifactorial etiology [**4**].

Hip trauma is another cause of secondary hip arthrosis in a young patient. This usually occurs in the setting of a motor vehicle accident, and may take the form of traumatic hip dislocation, proximal femoral fracture, or acetabular fracture. Long-term prognostic factors include the following: the severity of the lesion, the type and location of the fracture, and the presence or absence of secondary lesions (articular incongruence, femoral head necrosis) [**4**,**5**].

Slipped capital femoral epiphysis, with little understood etiology, often presents as hip pain in a child, predominantly male. It can evolve in time towards secondary hip arthrosis [**6**].

Femoral head necrosis is a redutable problem for the orthopedic surgeon, with an incidence hard to evaluate in the incipient stages. The etiology may be traumatic or non-traumatic, in which cases the causes may include: chronic alcohol abuse, steroid use [**7**], Gaucher’s disease, Cushing’s disease, and decompression sickness [**4**]. In all cases, the pathologic process consists in an interruption in the blood flow to the anterolateral region of the femoral head, leading to articular dysfunction and secondary hip arthrosis.

Legg-Calve-Perthes’ disease affects the infant, and consists of femoral head ischemia, leading to osteonecrosis and later on to hip arthrosis. Also, the necrotic area often involves the growth cartilage, which leads to abnormal ossification and growth, which may in turn lead to subchondral fractures, cartilage collapse, or limb length discrepancy [**4**].

Juvenile rheumatoid arthritis is a diagnosis at which we arrived by elimination, it affects girls more than boys, and is the most frequent cause of juvenile arthritis. The pathological process involves chronic synovial inflammation that leads to progressive articular destruction, with a lack of articular space with a varying degree of subchondral sclerosis, while the femoral head takes on a flattened look [**4**].

Hip joint infection leads to cartilage destruction in a short period of time. Long-term complications depend on several factors, the most important being the length of time that has passed until treatment has begun, the patient’s age, and the pathogen. One of the most important complications of hip infection is secondary hip arthrosis [**4**].

The treatment of hip arthrosis in a young patient may be conservative or surgical; conservative treatment aims to postpone the surgical intervention as much as possible and consists in pain medication, anti-inflammatory drugs, rest, and physical therapy.

Surgical treatment consists of either procedures that aim to preserve the patient’s own hip joint (hip arthroscopy; osteophytes excision associated with curettage and grafting of the acetabular cysts; proximal femoral or acetabular osteotomies; muscle release – the “hanging hip” procedure), or procedures that aim to replace the hip joint (total arthroplasty; hemiarthroplasty; resurfacing; arthrodesis). The most commonly used surgical procedure at this time is the total hip arthroplasty, followed by the resurfacing procedure.

Total hip arthroplasty involves the replacement of the acetabular surface and the femoral head with biologically inert implants, that are either impacted, or cemented into place, and have a polyethylene or ceramic insert at the interface between the implants. The femoral component may have a long or a short stem. The short femoral stem prosthesis is usually impacted in the proximal metaphysis of the femur, and it has a dedicated implantation technique and instruments, according to the manufacturer’s specifications. Currently, the most often used femoral short stem prostheses in our Clinic, are Proxima, Nanos and Tri-Lock.

## Case Presentation

**Fig. 1:**
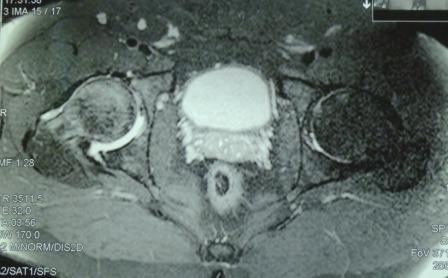
Preoperative MRI, showing the aseptic femoral head necrosis

We present the case of patient C. B., 23 years old at the time of the procedure, male, diagnosed with RIGHT HIP ARTHROSIS SECONDARY TO POSTCORTISONE ASEPTIC FEMORAL HEAD OSTEONECROSIS. The patient had been treated with cortisone injections for 6 months as treatment for vertebral disk pathology. His symptoms (pain and dysfunction of the hip) had been occurring for 18 months before diagnosis (**Fig. 1**). The decision was made to treat with total uncemented hip arthroplasty, opting for the use of the Proxima short femoral stem prosthesis, with a ceramic implant.

**Fig. 2:**
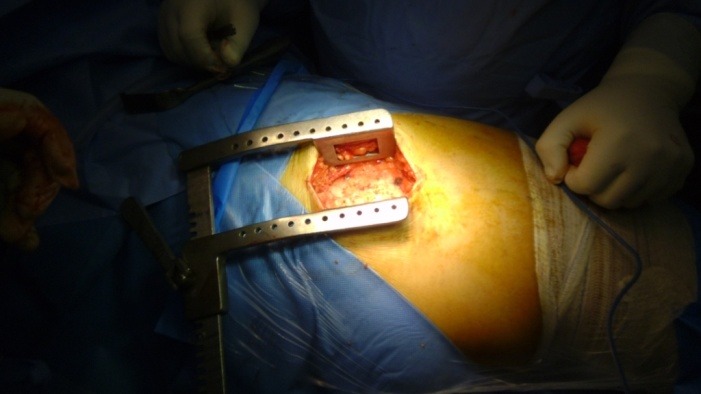
The minimally invasive posterolateral approach

**Fig. 3:**
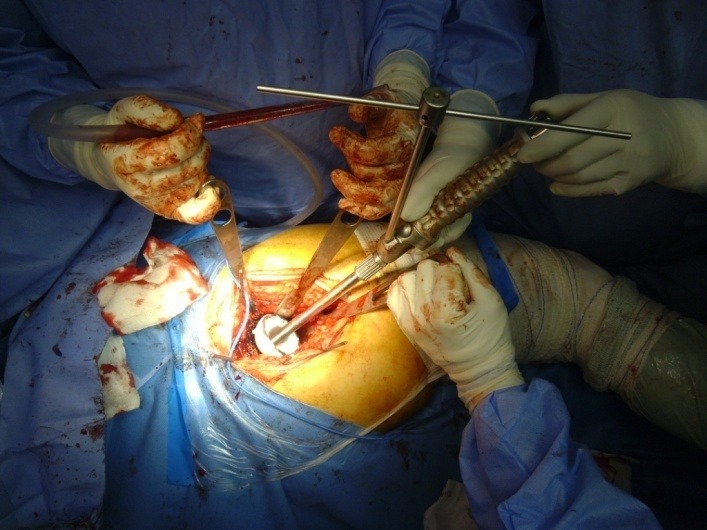
Surgical moment showing the impaction of the acetabular component

**Fig. 4:**
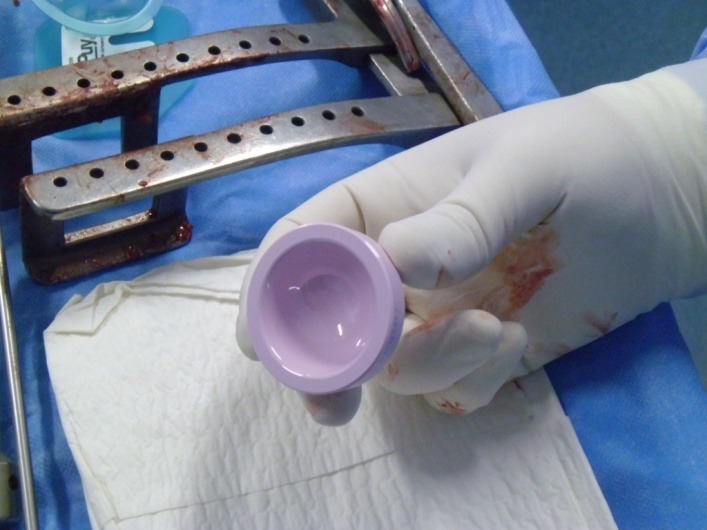
Surgical moment showing the ceramic insert

**Fig. 5:**
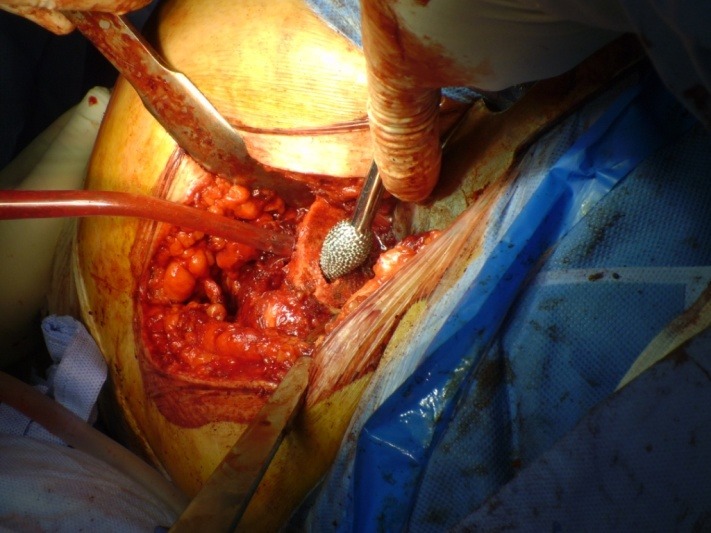
Surgical moment showing the preparation of the femoral metaphysis with dedicated instruments

**Fig. 6:**
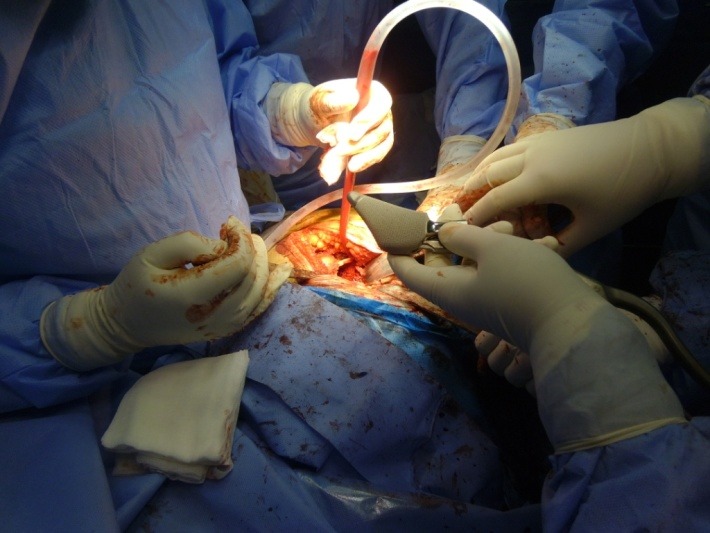
Surgical moment showing the Proxima prosthesis

**Fig. 7:**
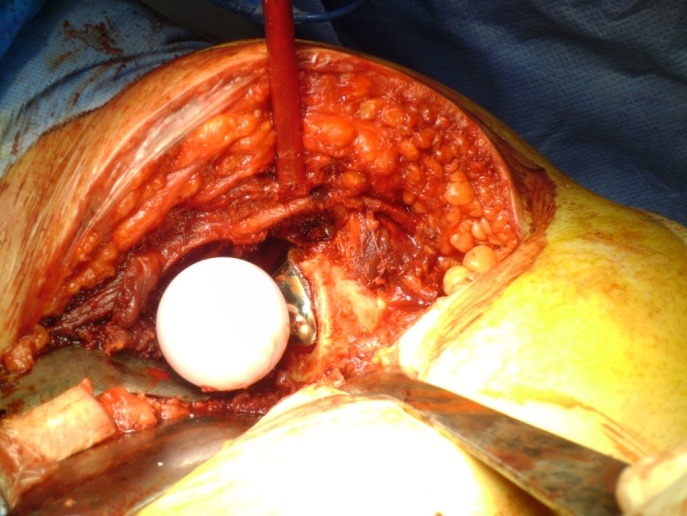
Surgical moment showing the ceramic femoral head

**Fig. 8:**
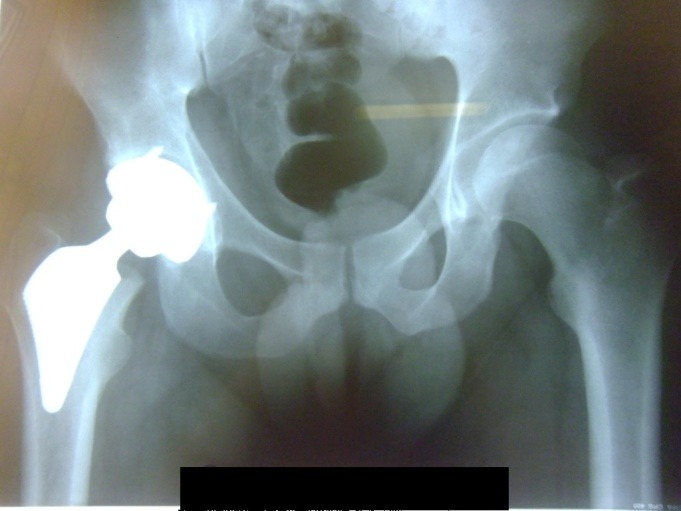
Postoperative X-Ray showing the implant

It was decided to use the minimally invasive posterolateral approach, with the patient in a lateral position, under spinal anesthesia (**Fig. 2**). The subcutaneous fat and the underlying fascia were dissected, and then a dissection through the muscle planes was done, releasing the short rotators. Afterwards, a “T” shaped incision in the articular capsule was exposed and made, after which the femoral head was dislocated and the neck osteotomy was performed. At this point the acetabulum was prepared, then, the final size was determined with trial cups and the acetabular component was impacted (**Fig. 3**). After impacting the acetabular component the ceramic insert was introduced (**Fig. 4**). The femoral neck and the metaphysis were prepared with dedicated instruments (**Fig. 5**), then the final size was determined and the Proxima short femoral stem prosthesis was impacted (**Fig. 6**). The femoral head size was determined and the adequate ceramic implant was impacted (**Fig. 7**), after which the prosthesis was reduced and tested with a range of motion maneuvers and impingement tests. The wound was actively drained and each anatomic layer, including the articular capsule, was sutured. 24 hours postoperatively the drains were removed and an X-ray was taken (**Fig. 8**). The patient’s evolution was favorable under postoperative antibiotics, antalgics, anti-inflammatory and antithrombotic drugs, associated with physical therapy, all according to our Clinic’s protocol, and the patient started near-full weight bearing at 24 hours.

## Discussion

The option was the short femoral stem hip arthroplasty because it has the advantage of conserving bone stock at the femoral neck, as it uses a high cervical osteotomy, which also conserves the natural femoral neck anteversion. This is important regarding the young patient, because a hip prosthesis, cemented or uncemented, has a limited time of use, needing multiple revisions that gradually decrease the local bone stock. The use of short stem prosthesis may delay by 15-20 years the revision with long femoral stm prosthesis; which will be more stabile if the local anatomy is distorted as little as possible.

Another advantage is linked with the possibility of implanting this prosthesis through a minimally invasive approach, which, although it is technically more demanding for the surgeon, is less traumatizing for the patient, and can be converted at any time to the classical approach, if necessary. Still, after the learning curve is surpassed, and with the help of dedicated instruments, the total time of the procedure may be brought down to the normal time of the classical uncemented hip arthroplasty, but with less blood lost, reduced local trauma, conserved bone stock, and a much smaller scar. The less surgical trauma means a faster recovery, less pain, and less time to a complete return to normal life.

At this time, three different short femoral stem prostheses are used in our Clinic: Proxima, Nanos, and Tri-Lock. These are all part of the same class of prosthetic components, and have both similarities, and notable differences.

The Proxima System may be used in primary hip arthroplasty, in a patient with satisfactory proximal femoral bone mass, in whom we wish to preserve that bone mass, to lessen the surgical trauma, and to load the femur proximally, in the metaphysis. In our Clinic, the system is used when performing arthroplasty on a young patient, but in effect the indication can be extended to any patient who has an indication for uncemented arthroplasty of the hip. Contraindications may include certain severe dysplasias of the hip joint, osteoporosis, hip osteotomies and femoral fractures in the patient’s history. The prosthesis must be implanted with a dedicated set of instruments. The special implantation “Round the Corner” technique permits, especially when used with a minimally invasive approach, to minimize the surgical trauma on the tissues.

The Nanos prosthesis allows for a metaphyseal fixation and necessitates a minimal bone resection at this level, preserving the bone mass for future revision surgery. The manufacturer gives the surgeon a choice between 10 sizes for the implant, and a dedicated set of instruments. Indications include primary and secondary hip arthrosis, hip dysplasia, avascular and posttraumatic femoral head necrosis, and an active patient under the age of 60. Contraindications include osteoporosis, coxa valga and coxa vara with a large angle, low body mass and past surgery in the local area.

The Tri-Lock system permits a superior initial stabilization, through its porous coating; which permits a biologic integration of the prosthesis. The shape of the prosthesis, with the femoral neck cut, allows the preservation of bone mass and improves the freedom of motion, while at the same time, lowering the risk of dislocation. The Tri-Lock prosthesis may be implanted either by a classical or by a minimally invasive approach.

## Conclusions

The short femoral stem prosthesis is a useful therapeutical option in the surgical pathology of the hip in the young patient, giving him a chance for an active life. This is the first step on the therapeutic surgical road, in time being succeeded by uncemented total hip arthroplasty, then by the uncemented one. This is important because each prosthesis has a limited average time of use, but at each revision, the bone stock is reduced, which may alter the indication for treatment. The use of the short femoral stem prosthesis allows a revision surgery that is easier to tolerate by the patient and is easier to perform by the surgeon.

There are several models of short stem femoral prosthesis, but no matter which one is chosen, the surgical indication, the surgical technique and a well-conducted recovery program are important. The surgeon must take into consideration the existence of the learning curve, and, in our experience, we recommend a firm grasp of the classic long femoral stem arthroplasty with the classic approach, as favored by each surgeon, before attempting to use the short femoral stem arthroplasty with a minimally invasive approach. The choosing of each arthroplastic implant must be made with care, taking into consideration the patient’s benefit, his expectations, and also the surgeon’s experience.

**Acknowledgements**

This work was supported by the staff in the Orthopaedics–Traumathology Clinic of ”Dr. Carol Davila” Central University Emergency Military Hospital in Bucharest, to whom we would like to extend our gratitude.
